# *BRCA1/2* Mutations in Vietnamese Patients with Hereditary Breast and Ovarian Cancer Syndrome

**DOI:** 10.3390/genes13020268

**Published:** 2022-01-29

**Authors:** Trong-Nhan N. Le, Van-Khanh Tran, Thu-Thuy Nguyen, Nam S. Vo, Tham H. Hoang, Hoang-Long Vo, Thanh-Hai T. Nguyen, Phuoc-Dung Nguyen, Viet-Tien Nguyen, Thanh-Van Ta, Huy-Thinh Tran

**Affiliations:** 1 Hanoi Medical University, Hanoi 100000, Vietnam; idoctor.spb@gmail.com (T.-N.N.L.); tranvankhanh@hmu.edu.vn (V.-K.T.); nguyenthuthuy@hmu.edu.vn (T.-T.N.); vohoanglonghmu@gmail.com (H.-L.V.); nguyenthanhhai@hmu.edu.vn (T.-H.T.N.); nvtien59@yahoo.com.vn (V.-T.N.); tathanhvan@hmu.edu.vn (T.-V.T.); 2 Center for Biomedical Informatics, Vingroup Big Data Institute, Hanoi 100000, Vietnam; v.namvs@vinbigdata.org (N.S.V.); v.thamhh@vinbigdata.org (T.H.H.); 3 National Institute of Hematology and Blood Transfusion, Hanoi 100000, Vietnam; dung.nguyenphuoc@gmail.com; 4 Hanoi Medical University Hospital, Hanoi Medical University, Hanoi 100000, Vietnam

**Keywords:** *BRCA1*, *BRCA2*, hereditary breast and ovarian cancer, NGS, Vietnam

## Abstract

(1) Background: Individuals with *BRCA1/2* gene mutations are at increased risk of breast and ovarian cancer. The prevalence of *BRCA1/2* mutations varies by race and ethnicity, and the prevalence and the risks associated with most *BRCA1/2* mutations has not been unknown in the Vietnamese population. We herein screen the entire *BRCA1* and *BRCA2* genes for breast and ovarian cancer patients with a family history of breast cancer and ovarian cancer, thereby, suggesting a risk score associated with carrier status and history for aiding personalized treatment; (2) Methods: Between December 2017 and December 2019, Vietnamese patients who had a pathological diagnosis of breast and epithelial ovarian cancer were followed up, prospectively, after treatment from two large institutions in Vietnam. Blood samples from 33 Vietnamese patients with hereditary breast and ovarian cancers (HBOC) syndrome were collected and analyzed using Next Generation Sequencing; (3) Results: Eleven types of mutations in both *BRCA1* (in nine patients) and *BRCA2* (in three patients) were detected, two of which (BRCA1:p.Tyr1666Ter and BRCA2:p.Ser1341Ter) have not been previously documented in the literature. Seven out of 19 patient’s relatives had *BRCA1/2* gene mutations. All selected patients were counselled about the likelihood of cancer rising and prophylactic screening and procedures. The study established a risk score associated with the cohorts based on carrier status and family history; (4) Conclusions: Our findings suggested the implications for the planning of a screening programme for *BRCA1* and *BRCA2* genes testing in breast and ovarian cancer patients and genetic screening in their relatives. *BRCA1/2* mutation carriers without cancer should have early and regular cancer screening, and prophylactic measures. This study could be beneficial for a diverse group in a large population-specific cohort, related to HBOC Syndrome.

## 1. Introduction

Among the malignancies in women, breast and ovarian cancers account for a high proportion of new cases and deaths. In a recent update on the global cancer burden, using the GLOBOCAN 2020, 2.3 million women with breast cancer and 3000 women with ovarian cancer were diagnosed in 2020 [1]. Vietnam, a lower middle-income country with over 97 million people, has one of the lowest age-standardized incidence rates for breast and ovarian cancer. The incidence rates of breast cancer is calculated to be 34.2 cases per 100,000 population per year, and of ovarian cancer is 2.4 per 100,000 population per year [1]. To the best of our knowledge, there is no effective screening method or national screening programme for ovarian cancers in Vietnam, therefore the identification of high-risk subjects of ovarian cancer for prevention and timely treatment is meaningful and necessary. One of the risk factors for breast and ovarian cancers is mutations of breast cancer susceptibility genes, which were known to be *BRCA1* and *BRCA2*—the dynamic regulators of genomic integrity. *BRCA1* and *BRCA2* inherited gene mutations, which greatly increase the risk of developing breast and ovarian cancers, are mutations that are frequently observed in hereditary breast and ovarian cancers (HBOC).

The detection of *BRCA1/2* mutations benefits both patients with HBOC and their family members. Patients benefit from platinum based chemotherapy and targeted treatment with Poly (ADP-ribose) polymerase (PARP) inhibitors [2], while unaffected family members may benefit from genetic counseling for HBOC, thereby obtaining timely screening and reducing surgery such as prophylactic mastectomy or salpingo-oophorectomy [3]. Although two studies of *BRCA1/2* gene mutations in the Vietnamese population were reported solely on patients themselves or in breast cancer [4,5], there have been no evidence on the mutations of these genes in HBOC patients. To date, there has been no official practice guideline for the diagnosis of HBOC syndrome in Vietnam; however, available national guidelines for the treatment of breast and ovarian cancer recommend target therapy for these patients with *BRCA1/2* gene mutations. To better understand the prevalence of the mutations in *BRCA1* and *BRCA2* genes among those with HBOC syndrome, this study was conducted to screen entire *BRCA1* and *BRCA2* genes for breast and ovarian cancer patients with a family history of breast cancer and ovarian cancer, thereby suggesting a risk score associated with carrier status and history for aiding personalized treatment.

## 2. Materials and Methods

### 2.1. Patients and Sample Collection

Between December 2017 and December 2019, Vietnamese patients who had a pathological diagnosis of breast and epithelial ovarian cancer were followed up, prospectively, after treatment at the National Hospital of Obstetrics and Gynecology (NHOG), and National Cancer Hospital. Family history and clinical data (gender, birthplace, age at cancer diagnosis, tumor type, immunohistochemistry and histology data) of the patients were obtained from medical records. Of them, 33 eligible patients met one of two indicated criteria for HBOC genetic analysis of (i) breast and ovarian cancer in the same individual diagnosed at any age, and/or (ii) two or more breast/ovarian cancer cases diagnosed in family members.

Prior to the collection of blood samples and clinical data from all participants, all probands and their family members were counselled to fulfill informed consent to participate in the study, as required by the Vietnam Medical Ethics Council. All genetic analyses were performed at the Center for Gene-Protein Research, Hanoi Medical University (Hanoi, Vietnam).

Samples of 2 mL peripheral blood with EDTA anticoagulation were collected from the patients. Genomic DNA was extracted from peripheral blood samples using a commercial kit (Magjet Whole blood DNA Kit, Thermo Scientific, Dreieich, Germany) in strict accordance with the manufacturer’s instructions.

### 2.2. Genetic Testing

Regarding *BRCA* sequencing, Next Generation Sequencing was the method of choice to sequence and the entire coding region of both genes was covered for all probands.

Genomic DNA was used for library creation using the Ultra II FS library preparation kit (New England Biolabs, Ipswich, MA, USA). PCR products from the library creation step were hybridized with a mixture of probes specific for both entire coding regions of *BRCA1* and *BRCA2* genes with biotin binding. Dynabeads MyOne Streptavidin T1 Beads, supplied by Thermo Fisher, were used to capture the DNA fragmentations based on streptavidin-biotin interaction, according to the instructions of the xGen library hybridization kit (IDTDNA, Coralville, IA, USA). The captured DNA fragments were cloned for the second time by PCR to reach the required concentration for next-generation sequencing (10 nM). Then, fragments were denatured and sequenced using the Nextseq 500/550 High Output kit on the NextSeq 550 system (Illumina, San Diego, CA, USA).

Next-generation sequencing resulted in millions of DNA sequence pairs with the size of 75 nucleotides. Raw sequence data analysis, including BaseCalling, demultiplexing, and alignment to the standard human genome of the National Center for Biotechnology Information (NCBI), was performed using the Genome Analysis Toolkit (GATK) version 4.1. The entire software was written based on the optimization analysis method by the Broad Institute of Harvard and MIT, USA [6]. The analysis results recorded genetic variations of *BRCA1* and *BRCA2* genes in selected patients.

Sequence variants were described according to the Human Genome Variation Society (HGVS) nomenclature guidelines. Variants were confirmed using Sanger sequencing, with the BigDye Terminator v3.1 Cycle Sequencing Kit and the ABI PRISM 3500 Genetic Analyzer.

Common polymorphisms represented 5% of the general population, and variants of uncertain significance (VUS) or pathogenic variants were then classified referring to the following databases: Breast Cancer Information Core BIC (https://research.nhgri.nih.gov/; accessed date: 7 July 2021), Clinical Variants (https://www.ncbi.nlm.nih.gov/pubmed; accessed date: 8 July 2021), Leiden Open (source) Variation Database (LOVD) (http://www.lovd.nl/3.0/home; accessed date: 8 July 2021), as well as population databases and data from clinicians, clinical laboratories, and researchers worldwide.

### 2.3. Statistical Analysis

#### 2.3.1. Survival Analysis for Breast Cancer and Ovarian Cancer Patients

Model fitting was performed using survival analysis of breast and ovarian cancer occurrences in the combined set of families. Individuals were censored at the age of occurrence in breast cancer and ovarian cancer. The model was adjusted to the mean observed age, with a distribution of the predictions around this age. Therefore, a subtraction was needed to obtain the estimated starting age of the study. We calculated interval censoring by subtracting cancer occurrence age by 30 without losing the generality [7,8,9].

#### 2.3.2. Breast and Ovarian Risk Prediction

We calculated a risk score based on the Breast and Ovarian Analysis of Disease Incidence and Carrier Estimation Algorithm (BOADICEA) [10,11]. The breast cancer incidence for individual *i*th at age t was assumed to be birth-cohort specific, and to depend on the underlying *BRCA1* or *BRCA2* genotype and polygenotype through a model of the form.

(1) $$\lambda_{i}\left(t\right)=\lambda_{k,0}\left(t\right)exp\left(X\right)$$

where k=0, 1, 2 for noncarriers, *BRCA1* and *BRCA2* carriers, respectively. X is a polygenic component, assumed to be normally distributed with a mean of 0 and variance of $\sigma^{2}$ and independent of age. The baseline incidence rates $\lambda_{k,0}\left(t\right)$ are chosen so that the overall age-specific incidence for both diseases, averaged over all genotypes, is constrained to agree with population incidence rates for England and Wales over the period 1983–1987 (Antoniou et al., 2002, Antoniou et al., 2004) [12,13]. Ovarian and breast cancer risks are assumed to be independent from given genotype. In this paper, we computed the probability that a woman of a given age x years developed breast or ovarian cancer by age x+n, given the known family history (*FH*). For example, if we denote the event of breast cancer by BC and the event of being unaffected by U, a generalized model can be formed as:

(2) $$P\left(Uatx,FH\right)=\frac{P\left(BCbtw\left(x,x+n\right),FH\right)}{P\left(Uatx,FH\right)}=\frac{\sum_{k=0}^{n-1}\lambda \left(x+k\right)S^{b}\left(x+k\right)S^{o}\left(x+k\right)}{P\left(Uatx,FH\right)}$$

The risks can be computed as the ratio of the likelihood of observing the pedigree, with the individual being unaffected at age x to the likelihood of observing mutated genes in the family. λ(x) is the breast cancer incidence at age x, and $S^{b}\left(t\right)$ and $S^{o}\left(t\right)$ are the probabilities of remaining free of breast and ovarian cancer respectively by age t. The probability was estimated based on the density of the incident rate given carrier status (e.g., z-score).

#### 2.3.3. Breast Cancer Familial Relative Risks

To estimate the polygenic effect (X in Equation (1)), it was assumed to be normally distributed with mean zero and variance $\sigma^{2}\left(t\right)$. We also incorporated into an age-specific familial relative risks (FRRs), polygenic effect, and carrier status for an individual with an affected family member (FM), such as mother, daughter, aunt and/or grandmother, which was predicted according to the below equation.

(3) $$\sigma^{2}\left(t\right)=\alpha +\beta_{1}t+\beta_{2}carrier+\beta_{3}\left(NumberofFMaffected\right)$$

## 3. Results

### 3.1. Detection of BRCA1 and BRCA2 Variants

A total of 33 women with HBOC syndrome were enrolled in this study. The mean age of the patients at diagnosis was 53.55 years (interquartile range, 34 to 80). Duplex cancer was present in six patients, 25 patients had only one type of cancer but had relatives diagnosed with ovarian/breast cancer, and two had both factors (Table 1).

The *BRCA1* and *BRCA2* panel used in this study covers all targeted coding exons and exon-intron boundaries. Eleven mutations in *BRCA1* and *BRCA2* genes were detected in 12 patients (36.4%), containing 8 *BRCA1* and 3 *BRCA2* pathogenic variants (Table 2) [14,15,16,17,18,19,20,21,22]. The BRCA1:c.4997dupA mutation was found in two patients. BRCA1:c.4997dupA and BRCA2:c.4022delC were detected for the first time in the current study. 

Eight mutations in the *BRCA1* gene comprised of three nonsense mutations (c.1621C > T, c.4997dupA, and c.5251C > T), three frameshift mutations (c.1016delA, c.2760_2763delACAG and c.5335delC), one missense mutation (c.5068A > C) and one splice site mutation in intron 16 (c.4986 + 4A > T).

**Figure 1:**
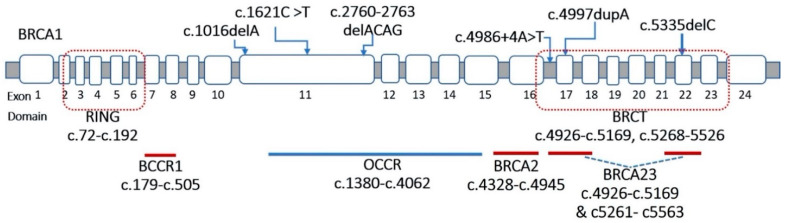
The location of the variants in the *BRCA1* gene.

In which, two mutations (c.1621C > T and c.2760_2763delACAG) located in an ovarian cancer cluster region (OCCR), four mutations (c.4986 + 4A > T, c.4997dupA, c.5068A > C and c.5335delC) in a breast cancer cluster region (BCCR). All BCCR mutations were located in the BRCT domain, which was the BACH1 binding site (Figure 1). 

**Figure 2:**
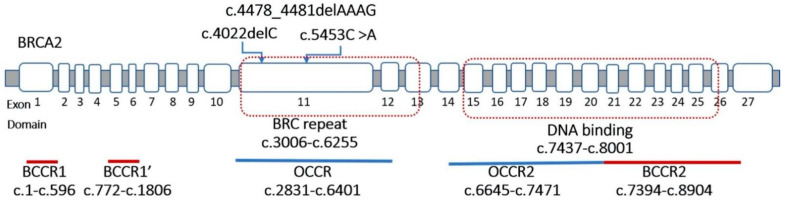
The location of the variants in the BRCA2 gene.

Three mutations in the *BRCA2* gene included two nonsense mutations (c.4022delC and c.5453C > A) and one frameshift mutation (c.4478_4481delAAAG). All of these mutations were located in exon 11 of the OCCR, or BRC repeat region, which was the binding site of protein RAD51 (Figure 2).

**Figure 3:**
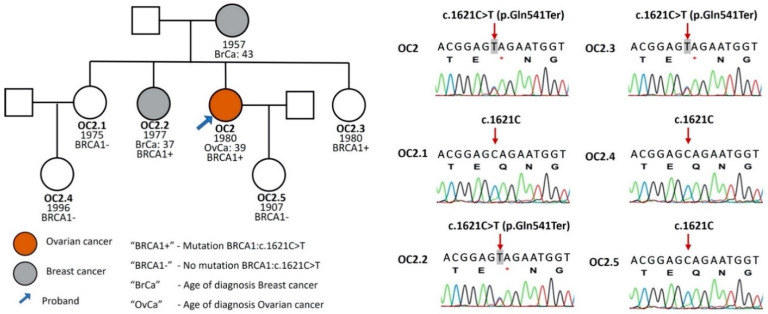
Pedigree and sequencing results of the family with mutation BRCA1:c.1621C > T. Grey—breast cancer. Array—patient in the study. “BRCA1+”—patients with *BRCA1* mutation. “BRCA1”—patients with no mutation of *BRCA1*.

Blood samples from the family members of the 19 women who had *BRCA1/2* mutations were collected in order to perform a genetic test. Seven of them (36.8%) were found to have *BRCA1/2* gene mutations. A pedigree chart is shown in Figure 3.

### 3.2. Survival Analysis for Breast Cancer and Ovarian Cancer Patients

**Figure 4:**
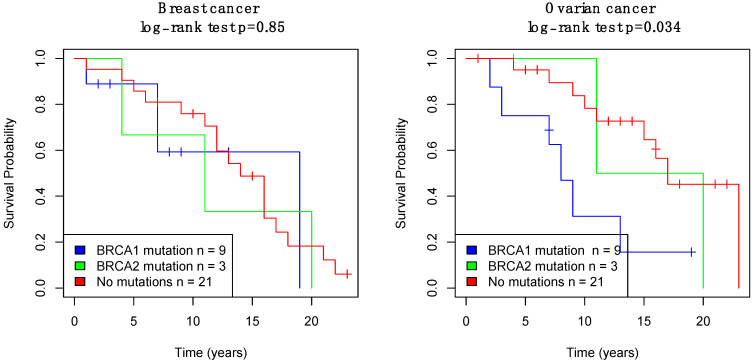
Survival analysis on breast cancer and ovarian cancer of the dataset based on patients with *BRCA1/2* mutation and patients with no mutation.

The patients with *BRCA1* mutation had the worst survival in both breast cancer and ovarian cancer, compared to the patients with *BRCA2* mutation and patients with no mutations. The separation in ovarian cancer among patient groups was observed to be statistically significant (log-rank test *p* = 0.034), while there was no statistically significant difference in survival among patients with *BRCA1/2* mutation and no mutations in breast cancer (log-rank test *p* = 0.85) (Figure 4). Since the number of patients in each group was low, the survival analysis suggested that the trend of having a mutation of the *BRCA1/2* gene or not could impact overall survival in breast cancer and ovarian cancer patients.

### 3.3. Cancer Risks

**Figure 5:**
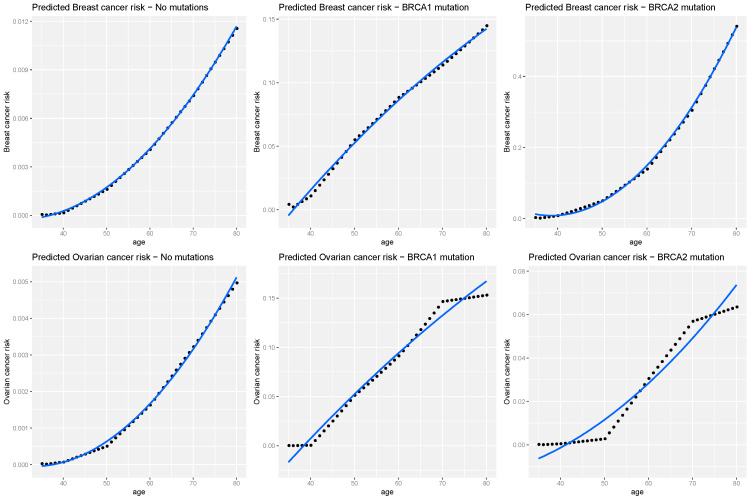
Predicted risks of breast cancer and ovarian cancer between 35 to 80 years, to a 34-year-old female given no FH of breast and/or ovarian cancer. Blue line and the shaded grey area fit the curves and 95% confidence interval. Blueline and the shaded grey area fit the curves and 95% confidence interval in smoothed line for incident rates at each age.

The dataset showed that 8/33 (~24%) had both cancers, with the equation needing to consider both $S^{b}\left(t\right)$ and $S^{o}\left(t\right)$ for the additional risk for each cancer. Our analysis indicated that the individual woman’s risk increased overtime, regardless of mutation status. However, when a mutation occurred, the risk changed rapidly with *BRCA1* mutation patients (middle plots) (Figure 5). In this plot, the incident rate of the cohort was used to predict the risk for each age, from 34 to 80. The rate was smoothed by a regression with a 95% confidence interval. 

### 3.4. Breast Cancer Familial Relative Risks

**Figure 6:**
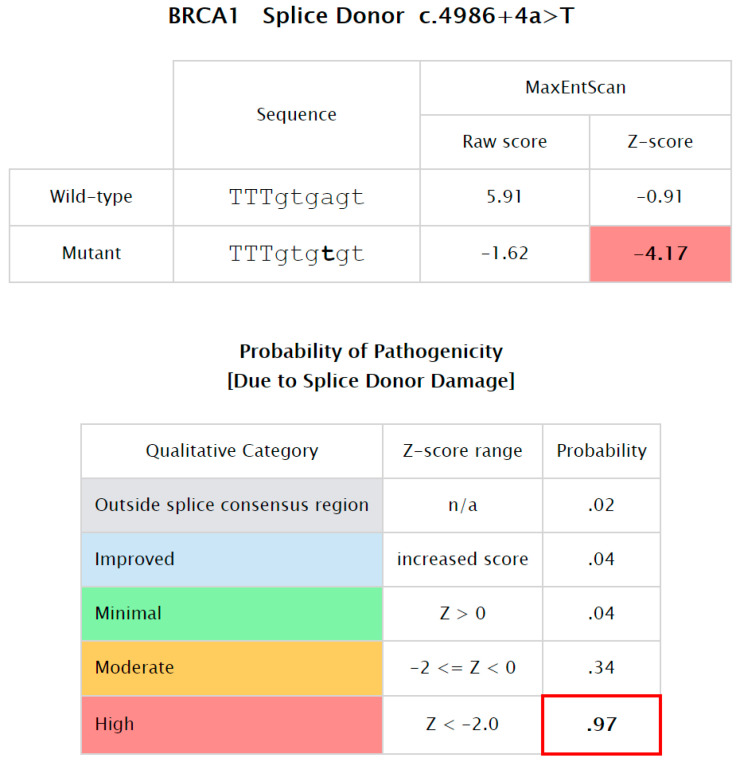
Probaility of Pathogenicity of BRCA1:c.4986 + 4A > T.

Our analysis showed that there were no significant predictors for the *BRCA* familial relative risks. Thus, to estimate the effect of the carrier and family history, a larger cohort size and a more developed follow-up scheme are needed. The splicing-level estimation was due to splice donor damage, which introduced a high prior probability of pathogenicity (0.97) by using in Silico Prior Probability of Pathogenicity from the HCI Breast Cancer Genes Prior Probabilities website (Figure 6).

## 4. Discussion

While the presence of mutations in the *BRCA1* and *BRCA2* genes have been characterized in many populations in North America and Europe since 1994, the prevalence of these mutations has been, in recent years, estimated for a number of Asian populations such as China, Japan, Korea, the Philippines, Thailand, and Malaysia. In Vietnam, Ginsburg (2010) firstly found two (0.8%) *BRCA1/2* gene mutations among 259 selected breast cancers in women [23]. Here, we report detailed research about *BRCA1/2* gene mutations among HBOC patients in Vietnam. With two HBOC criteria in the current study, *BRCA1/2* gene mutations were detected in 12 out of 33 patients. In the database of Genetic Center Myriad, USA by Frank (2002), estimating model risk of *BRCA1/2* mutation, individuals with two criteria HBOC had about 4–64% risk of *BRCA1/2* mutation [24].

We found no significant difference in the mean age at diagnosis amongst patients with *BRCA1/2* mutation and non-mutation (*t*-test *p* = 0.391). Mean age at diagnosis was younger in *BRCA1* mutation patients (48 years) compared to in *BRCA2* mutation carriers (61 years). The present result was consistent with previous findings such as that ovarian cancer onset in *BRCA2* mutation patients was an average 8-10 years later than in patients with *BRCA1* mutation [25].

All identified mutations were checked for clinical significance in *BRCA* exchange. There were five nonsense mutations (c.1621C > T, c.4997dupA, c.5251C > T in *BRCA1* and c.4022delC, c.5453C > A in *BRCA2*), where deleted, replaced, or duplicated single nucleotide were found, in addition to protein level, changing amino acid to a premature termination codon. They were predicted to cause loss of normal protein function through either protein truncation or nonsense-mediated mRNA decay. This is why their clinical significance is determined or predicted as pathogenic. Although the *BRCA2*: c4022delC mutation was first found in our study, its protein level change p.Ser1341Ter is similar to the *BRCA2*:c4022C > A mutation, which was confirmed as pathogenic.

Four frameshift mutations (c.1016delA, c.2760_2763delACAG and c.5335delC in *BRCA1* and c.4478_4481delAAAG in *BRCA2*), with deleted or some nucleotide, made changes in normal sequence after mutation positions. All created premature stop codons of the new reading frames were predicted to cause a truncated or absent protein, which are commonly known mechanisms for disease. All of them had pathogenic significance.

The current results found one missense mutation *BRCA1*: c.5068A > C, where nucleotide A was replaced by C and at the protein level changed a Lysine to a Glutamine (AAA > CAA) and did not create a premature termination codon. There are conflicting interpretations of pathogenicity between likely benign and uncertain significance. We consider it to be a variant of uncertain significance.

Only one splice-site mutation c.4986 + 4A > T in the 16th intron of *BRCA1* did not directly change the encoded amino acid sequence of the *BRCA1* protein, but it affected a nucleotide within the consensus splice site of the intron. Experimental studies indicated that this variant disrupted mRNA splicing and/or protein function [26]. The splicing-level estimation is due to splice donor damage, which introduces a high prior probability of pathogenicity (0.97) by using in Silico Prior Probability of Pathogenicity from the HCI Breast Cancer Genes Prior Probabilities website (Figure 6). In addition, a different nucleotide that changed at the same position (c.4986 + 4A > T) was reported as a pathogenic intronic splicing mutation in a cohort of 585 Slovak individuals with family histories of breast and ovarian cancer [17].

Depending on the location of mutation, Rebbeck et al., (2015) suggested some OCCRs and BCCR, where mutations had different risks of ovarian and breast cancer [27]. In our study, five mutations (in *BRCA1* and in *BRCA2*) were OCCRs, which means they had higher ovarian cancer risk and lower breast cancer risk than mutations in other regions. In addition, these mutations in BCCR also lie in the domain of the BRCT of the *BRCA1* gene, where protein BACH1 and other functional proteins bind to interact with protein *BRCA1*. Based also on analyzing the type and location of mutation, Rebbeck’s study revealed that all nonsense mutations in exon 11 of both genes had higher ovarian cancer risk and lower breast cancer risk compared to mutations at other regions and/or with other types [27].

In a recent Vietnam report of Hoang Anh Vu (2020), there were four types of pathogenic *BRCA1* mutations in Vietnamese patients with ovarian cancer. Among these patients, *BRCA1*: c.1621C > T was present in two patients, *BRCA1*: c.5251C > T in four patients and *BRCA1*: c.5335delC in one case [4]. In a study of 200 cases of Asian origin on breast cancer patients living in the USA, three in four Vietnamese patients were identified with the mutation c.5251C > T in *BRCA1* [28]. BRCA1:c.4997dupA in our study was detected in one ovarian cancer patient and one breast cancer patient, and they both had one daughter with this mutation. Whether four mutations (c.1621C > T, c.5251C > T c.4997dupA, c.5251C > T and c.5335delC) in *BRCA1* gene are “founder mutations”, predisposing breast and ovarian cancer in the Vietnamese cohort, this needs to be further studied with a larger patient sample size.

Our patients were informed about the detected gene mutations, which was necessary for their oncologists to consider the use of suitable targeted therapy with PARP inhibitors. Seven in 19 relatives of patients who had *BRCA1/2* gene mutations received genetic counseling for the risk of ovarian and breast cancer, in addition to screening methods and prophylactic measures according to the recommendation of the National Comprehensive Cancer Network (NCCN) [29]. We developed a risk score that took into account the risk of the patients with *BRCA1/2* mutation and non-mutation and family history based on BOADICEA from the University of Cambridge (UK), contributing to further determine the HBOC risk in the future. Even though the incident rates were estimated based on a UK population, it can be gradually changed in the context of further updated studies in a Vietnamese population.

## 5. Conclusions

This was the first study in Vietnam to investigate mutations of *BRCA1* and *BRCA2* genes in breast and ovarian cancer patients with the criteria of HBOC syndrome, and to develop a risk score associated with the cohort based on carrier status and family history. *BRCA1* gene mutations were present in 27.3% patients, while *BRCA2* gene mutations were in 9.1% patients. Notably, two novel mutations (BRCA1: c.4997dupA and BRCA2: c.4022delC) were first documented in our study. Current findings suggested implications for the planning of a screening programme for *BRCA1* and *BRCA2* genes testing in breast and ovarian cancer patients and genetic screening of their relatives. Based on mutation identification, we can individualize treatment, prognosis, and follow-up more effectively, especially for Poly ADP ribose polymerase enzyme inhibitor therapy. Finally, patients with *BRCA1/2* mutations without cancer should also receive early and regular cancer screening, and prophylactic measures.

## Figures and Tables

**Table 1:** Patients with hereditary breast and ovarian cancer syndrome.

Characteristics	Patients with *BRCA1/2* Mutations			Non-Mutation Patient	Total
	*BRCA1* Mutation Patient	*BRCA2* Mutation Patient	Total		
	N = 9 (27.3%)	N = 3 (9.1%)	N = 12 (36.4%)	N = 21 (64.6%)	N = 33 (100%)
Mean of diagnosed age (years)	48.0	61.0	51.25	54.86	53.55
HBOC					
FH (OC and/or BC) only	8	1	9	16	25
Duplex cancer only	0	2	2	4	6
Both (FH & Duplex cancer)	1	0	1	1	2

FH: family history for breast and/or ovarian cancer; BC: previous breast cancer history; OC: ovarian cancer.

**Table 2:** List of *BRCA1/2* genes variants.

No.	HGVS cDNA	HGVS Protein	Local	BCCR/ OCCR	Domain /Binding	Type	Sign.	Ref.
01	c.1016delA	p.Lys339ArgfsTer2	BRCA1-Exon 11	−	−	F	P	[13]
02	c.1621C > T	p.Gln541Ter	BRCA1-Exon 11	OCCR1	−	N	P	[14]
03	c.2760_2763delACAG	p.Thr922LeufsTer77	BRCA1-Exon 11	OCCR3	−	F	P	[15]
04	c.4986 + 4A > T	IVS16+4A > T	BRCA1-Intron 16	BCCR23	BRCT/BACH1	Ss	P	[16]
05	c.4997dupA	p.Tyr1666Ter	BRCA1-Exon 17	BCCR23	BRCT/BACH1	N	P	CS
06	c.5068A > C	p.Lys1690Gln	BRCA1-Exon 17	BCCR23	BRCT/BACH1	M	VUS	[17]
07	c.5251C > T	p.Arg1751Ter	BRCA1-Exon 20	−	−	N	P	[18]
08	c.5335delC	p.Gln1779AsnfsTer14	BRCA1-Exon 22	BCCR23	BRCT/BACH1	F	P	[19]
09	c.4022delC	p.Ser1341Ter	BRCA2-Exon 11	OCCR1	−	N	P	CS
10	c.4478_4481delAAAG	p.Glu1493ValfsTer10	BRCA2-Exon 11	OCCR1	−	F	P	[20]
11	c.5453C > A	p.Ser1818Ter	BRCA2-Exon 11	OCCR1	−	N	P	[21]

OCCR: ovarian cancer cluster region; BCCR: breast cancer cluster region; F: frameshift; N: nonsense; M: missense; Ss: splice site; (−): absent; P: pathogenic; VUS: variant uncertain significance; CS: current study.

## Data Availability

The data used in this article are available upon request from the corresponding author. The data are not publicly available due to the protection of personal data.

## References

[1] Sung H., Ferlay J., Siegel R.L., Laversanne M., Soerjomataram I., Jemal A., Bray F. (2021). Global Cancer Statistics 2020: GLOBOCAN Estimates of Incidence and Mortality Worldwide for 36 Cancers in 185 Countries. CA Cancer J. Clin..

[2] Neff R.T., Senter L., Salani R. (2017). BRCA mutation in ovarian cancer: Testing, implications and treatment considerations. Ther. Adv. Med. Oncol..

[3] Finch A.P., Lubinski J., Møller P., Singer C.F., Karlan B., Senter L., Rosen B., Maehle L., Ghadirian P., Cybulski C. (2014). Impact of oophorectomy on cancer incidence and mortality in women with a BRCA1 or BRCA2 mutation. J. Clin. Oncol..

[4] Vu H.A., Phu N.D., Khuong L.T., Hoa P.H., Nhu B.T.H., Nhan V.T., Thanh L.Q., Sinh N.D., Chi H.T., Quan N.D. (2020). Recurrent BRCA1 Mutation, but no BRCA2 Mutation, in Vietnamese Patients with Ovarian Carcinoma Detected with Next Generation Sequencing. Asian Pac. J. Cancer Prev..

[5] Van Thuan T., Van Chu N., Khoa P.H., Quang N.T., Van Tu D., Tho N.T.Q., Huyen P.T., Ha B.H., Han P.T., Long D.M. (2020). A Novel BRCA1 Gene Mutation Detected With Breast Cancer in a Vietnamese Family by Targeted Next-Generation Sequencing: A Case Report. Breast Cancer.

[6] McKenna A., Hanna M., Banks E., Sivachenko A., Cibulskis K., Kernytsky A., Garimella K., Altshuler D., Gabriel S., Daly M. (2010). The Genome Analysis Toolkit: A MapReduce framework for analyzing next-generation DNA sequencing data. Genome Res..

[7] Peto J., Collins N., Barfoot R., Seal S., Warren W., Rahman N., Easton D.F., Evans C., Deacon J., Stratton M.R. (1999). Prevalence of BRCA1 and BRCA2 gene mutations in patients with early-onset breast cancer. J. Natl. Cancer Inst..

[8] Tilanus-Linthorst M.M., Lingsma H.F., Evans D.G., Thompson D., Kaas R., Manders P., van Asperen C.J., Adank M., Hooning M.J., Kwan Lim G.E. (2013). Optimal age to start preventive measures in women with BRCA1/2 mutations or high familial breast cancer risk. Int. J. Cancer.

[9] van der Groep P., Bouter A., van der Zanden R., Siccama I., Menko F.H., Gille J.J.P., van Kalken C., van der Wall E., Verheijen R.H.M., van Diest P.J. (2006). Distinction between hereditary and sporadic breast cancer on the basis of clinicopathological data. J. Clin. Pathol..

[10] Antoniou A.C., Cunningham A.P., Peto J., Evans D.G., Lalloo F., Narod S.A., Risch A.H., Eyfjord J.E., Hopper J.L., Southey M.C. (2008). The BOADICEA model of genetic susceptibility to breast and ovarian cancers: Updates and extensions. Br. J. Cancer.

[11] Lee A., Mavaddat N., Wilcox A.N., Cunningham A.P., Carver T., Hartley S., de Villiers C.B., Izquierdo A., Simard J., Schmidt M.K. (2019). Correction: BOADICEA: A comprehensive breast cancer risk prediction model incorporating genetic and nongenetic risk factors. Genet. Med..

[12] Antoniou A.C., Pharoah P.D.P., McMullan G., Day N.E., Stratton M.R., Peto J., Ponder B.J., Easton D.F. (2002). A comprehensive model for familial breast cancer incorporating BRCA1, BRCA2 and other genes. Br. J. Cancer.

[13] Antoniou A.C., Pharoah P.P.D., Smith P., Easton D.F. (2004). The BOADICEA model of genetic susceptibility to breast and ovarian cancer. Br. J. Cancer.

[14] Hogervorst F.B., Cornelis R.S., Bout M., Van Vliet M., Oosterwijk J.C., Olmer R., Bakker B., Klijn J.G., Vasen H.F., Meijers-Heijboer H. (1995). Rapid detection of BRCA1 mutations by the protein truncation test. Nat. Genet..

[15] Debatin I., Tonin P., Royer-Pokora B., Dong J., Chang-Claude J., Wu Y., Schumacher V. (1998). A high proportion of mutations in the BRCA1 gene in German breast/ovarian cancer families with clustering of mutations in the 3′ third of the gene. Hum. Genet..

[16] van der Hout A.H., van den Ouweland A.M., van der Luijt R.B., Gille H.J., Bodmer D., Brüggenwirth H., Mulder I.M., van der Vlies P., Elfferich P., Huisman M.T. (2006). A DGGE system for comprehensive mutation screening of BRCA1 and BRCA2: Application in a Dutch cancer clinic setting. Hum. Mutat..

[17] Konecny M., Milly M., Zavodna K., Weismanova E., Gregorova J., Mlkva I., Ilencikova D., Kausitz J., Bartosova Z. (2011). Comprehensive genetic characterization of hereditary breast/ovarian cancer families from Slovakia. Breast Cancer Res. Treat..

[18] Ang P., Lim I.H., Lee T.-C., Luo J.-T., Ong D.C., Tan P.H., Lee A.S. (2007). BRCA1 and BRCA2 mutations in an Asian clinic-based population detected using a comprehensive strategy. Cancer Epidemiol. Biomarkers Prev..

[19] Vehmanen P., Friedman L.S., Eerola H., McClure M., Ward B., Sarantaus L., Kainu T., Syrjäkoski K., Pyrhönen S., Kallioniemi O.-P. (1997). Low proportion of BRCA1 and BRCA2 mutations in Finnish breast cancer families: Evidence for additional susceptibility genes. Hum. Mol. Genet..

[20] Matsuda M.L.D.L., Liede A., Kwan E., Mapua C.A., Cutiongco E.M.C., Tan A., Narod S.A. (2002). BRCA1 and BRCA2 mutations among breast cancer patients from the Philippines. Int. J. Cancer.

[21] Tavtigian S.V., Simard J., Rommens J.M., Couch F.J., Shattuck-Eidens D., Neuhausen S.L., Merajver S.D., Thorlacius S., Offit K., Stoppalyonnet D. (1996). The complete BRCA2 gene and mutations in chromosome 13q-linked kindreds. Nat. Genet..

[22] Zhao Q., Yang J., Li L., Cao D., Yu M., Shen K. (2017). Germline and somatic mutations in homologous recombination genes among Chinese ovarian cancer patients detected using next-generation sequencing. J Gynecol. Oncol..

[23] Ginsburg O., Dinh N., To T., Quang L., Linh N., Duong B., Royer R., Llacuachaqui M., Tulman A., Vichodez G. (2011). Family history, BRCA mutations and breast cancer in Vietnamese women. Clin. Genet..

[24] Frank T.S., Deffenbaugh A.M., Reid J.E., Hulick M., Ward B.E., Lingenfelter B., Gumpper K.L., Scholl T., Tavtigian S.V., Pruss D.R. (2002). Clinical characteristics of individuals with germline mutations in BRCA1 and BRCA2: Analysis of 10,000 individuals. J. Clin. Oncol..

[25] Powell C.B., Kenley E., Chen L.-M., Crawford B., McLennan J., Zaloudek C., Komaromy M., Beattie M., Ziegler J. (2005). Risk-reducing salpingo-oophorectomy in BRCA mutation carriers: Role of serial sectioning in the detection of occult malignancy. J. Clin. Oncol..

[26] Findlay G.M., Daza R.M., Martin B., Zhang M.D., Leith A.P., Gasperini M., Janizek J.D., Huang X., Starita L.M., Shendure J. (2018). Accurate classification of BRCA1 variants with saturation genome editing. Nature.

[27] Rebbeck T.R., Mitra N., Wan F., Sinilnikova O.M., Healey S., McGuffog L., Mazoyer S., Chenevix-Trench G., Easton D.F., Antoniou A.C. (2015). Association of type and location of BRCA1 and BRCA2 mutations with risk of breast and ovarian cancer. JAMA.

[28] Kurian A.W., Gong G.D., Chun N.M., Mills M.A., Staton A.D., Kingham K.E., Crawford B.B., Lee R., Chan S., Donlon S.S. (2008). Performance of BRCA1/2 mutation prediction models in Asian Americans. J. Clin. Oncol..

[29] NCCN (2017). NCCN Clinical Practice Guidelines in Oncology (NCCN Guidelines®) Genetic/Familial High-Risk Assecessment: Breast and Ovarian Version 2.

